# Nursing in the context of the COVID-19 pandemic: what lessons have we learned?^1^

**DOI:** 10.1590/0034-7167.2022750601

**Published:** 2022-07-08

**Authors:** Dulce Aparecida Barbosa, Janine Schirmer, Alexandre Pazetto Balsanelli

**Affiliations:** I Full Professor and Professor Habilitation. Department of Clinical and Surgical Nursing. Universidade Federal de São Paulo, Escola Paulista de Enfermagem. São Paulo, São Paulo, Brazil.; II Full Professor and Professor Habilitation. Department of Nursing in Women’s Health. Universidade Federal de São Paulo, Vice-Director at Escola Paulista de Enfermagem. São Paulo, São Paulo, Brazil.; III Adjunct Professor. Department of Administration in Health and Nursing Services. Universidade Federal de São Paulo, Director at Escola Paulista de Enfermagem. São Paulo, São Paulo, Brazil.

In celebration of International Nurses Day, celebrated on 05/12, we want to reflect in light of the central theme proposed by the Brazilian Nursing Association (ABEn - *Associação Brasileira de Enfermagem*) what lessons we learned from COVID-19. The COVID-19 pandemic, like all crises experienced by human beings, especially global crises, teaches everyone something. The planet’s nursing has mobilized in the fight against the disease, in the care of the sick and in preventive measures, as is part of the professional mister. This scenario of a health crisis leads us to some reflections: what has the pandemic taught our professional category? What foundations can we build for a better future for the profession, for professionals and for all social groups in our society? What lessons have been learned?

**Lesson 01:** it was possible to verify a huge gap between social groups, cruelly revealing the less favored, those considered the base of the socio-economic pyramid, the large mass of low-skilled workers and, therefore, workers without decent work. Added to this, it was possible to verify that the mantle of the Unified Health System (SUS – *Sistema Único de Saúde*) unequally covers these populations, i.e., it gives more blanket to those who have more strength to pull. The hard-won achievements over decades to comply with the legal provision “health as a State duty and a right for all...” were neglected. The delay in the decision to vaccinate and use this means, as well as in the laxity or leniency in following banal health standards, such as mask use and hand hygiene (which serve for any diseases spread through the air), has put thousands of people at the mercy of COVID-19. Nursing took care of everyone, without discrimination, but it was obvious that beds and personnel would be lacking to deal with a pandemic avalanche like this. Also in nursing, where, like the rest of society, there are groups that are more or less privileged in work processes, the pandemic hit hardest those with worse working conditions. There is still a lesson to be done with learning about inequalities: an overview of nursing actions and epidemiological mapping to assess the impact of the pandemic among different groups in our profession. Detected and assessed, transforming the necessary changes into rights for nursing professionals as a State public policy. The category is responsible for the systematic and continuous process of assessing working conditions, monitoring the workforce, instruments and safer conditions for the exercise of the profession.

**Lesson 02:** one of the most worrying phenomena detected during the pandemic was the significant increase in domestic violence, more specifically violence against women and children. This phenomenon extended to Brazilian nursing, performed mostly by women, and, to some extent, violence reached these professionals as well. Do we have data? Do we have numbers? Do we have ways to combat the violence suffered by nursing professionals? In the pandemic, we learned the lesson that monitoring, especially epidemiological monitoring, is important in order to be able to interfere early in the course of disease or health problems. What can we do to transfer this learning into data on Brazilian nursing? It is once again necessary to reinforce public policies and transform them into practices: each workplace with more nursing workers must have an office/channel for reporting violence, as well as support and follow-up to overcome it.

**Lesson 03:** qualify, qualify and qualify! The practice of nursing, like many professions, demands continuous improvement not only in the most current and impactful techniques and procedures, but also in terms of ethics and social commitment. Therefore, professional qualification is not only a market requirement, it is and should be a requirement of every professional to combat iatrogenic and continue offering scientifically based work; more than that, it must be a requirement that workplaces provide these systematic, in-depth and remunerated qualifying processes, i.e., within their working period. If educational institutions have the obligation to produce quality professionals with skills and knowledge, in addition to ethically committed attitudes, health service institutions must have the obligation to systematically and continuously improve the qualification processes of nursing workers.

Finally, in all these years of university education that Brazilian nursing has, and with a high number of doctors or nursing scientists, we are still influential in the definition of care policies. What is missing to have greater autonomy over nursing care, whether to assist the sick or to prevent diseases, transforming some universal principles of care into State policy? The pandemic has taught us a lot, but we have much more to learn from the correct assessment of our successes and mistakes, as these will help us better plan our actions for future demands as critical as the COVID-19 pandemic. At first, about the post-COVID-19 period, how to face the consequences, sequels and long side effects caused by the illness or by the treatment given in the illness? What kind of iatrogenic could our “urgent care for the unknown” have occurred? What instruments do we have or need to create to take care of those who have lost their entire family? Where will nursing professionals be welcomed and cared for in the aftermath of stressful years caring for and supporting patients and families? It will be necessary to develop post-pandemic care projects and ask this question again in a year or two. We have a lot of hope, because we are resilient, we will always exercise our profession under the foundation of science, ethics and with a lot of criticism.

## Long life to Nursing and to the profession!

Available from: https://sp.unifesp.br/comunicasp/noticias/a-enfermagem-no-contexto-da-pandemia-pela-covid-19-que-licoes-aprendemos
